# Data mining of digitized health records in a resource-constrained setting reveals that timely immunophenotyping is associated with improved breast cancer outcomes

**DOI:** 10.1186/s12885-018-4833-4

**Published:** 2018-09-27

**Authors:** Arturo López-Pineda, Mario F Rodríguez-Moran, Cleto Álvarez-Aguilar, Sarah M Fuentes Valle, Román Acosta-Rosales, Ami S Bhatt, Shruti N Sheth, Carlos D Bustamante

**Affiliations:** 1 Department of Biomedical Data Science, School of Medicine, Stanford University, 1265 Welch Road, Stanford, California 94305 USA; 2 Facultad de Ciencias Médicas y Biológicas “Dr. Ignacio Chávez”, Universidad Michoacana de San Nicolás de Hidalgo, Av. Dr. Rafael Carrillo S/N, Esq. Dr. Salvador González Herrejón, Morelia, 58020 Michoacán Mexico; 3 Coordinación de Investigación en Salud, Delegación Michoacán, Instituto Mexicano del Seguro Social, Av. Madero Poniente No. 1200, Morelia, 58000 Michoacán Mexico; 4 Hospital General Regional No. 1, Servicio de Ginecobstetricia, Instituto Mexicano del Seguro Social, Bosques de los Olivos No. 101, Charo, 61303 Michoacán Mexico; 5 Delegación Michoacán, Instituto Mexicano del Seguro Social, Av. Madero Poniente No. 1200, Morelia, 58000 Michoacán Mexico; 6 Department of Genetics, School of Medicine, Stanford University, 300 Pasteur Drive, Stanford, 94305 California USA; 7 Department of Medicine (Hematology), School of Medicine, Stanford University, 875 Blake Wilbur Drive, Stanford, 94305 California USA; 8 Department of Medicine (Oncology), School of Medicine, Stanford University, 875 Blake Wilbur Drive, Stanford, 94305 California USA

**Keywords:** Breast neoplasms, Immunohistochemistry, Tumor biomarkers, Medical records, Clinical coding, Data mining, Mexico

## Abstract

**Background:**

Organizations that issue guidance on breast cancer recommend the use of immunohistochemistry (IHC) for providing appropriate and precise care. However, little focus has been directed to the identification of maximum allowable turnaround times for IHC, which is necessary given the diversity of hospital settings in the world. Much less effort has been committed to the development of digital tools that allow hospital administrators to monitor service utilization histories of their patients.

**Methods:**

In this retrospective cohort study, we reviewed electronic and paper medical records of all suspected breast cancer patients treated at one secondary-care hospital of the Mexican Institute of Social Security (IMSS), located in western Mexico. We then followed three years of medical history of those patients with IHC testing.

**Results:**

In 2014, there were 402 breast cancer patients, of which 30 (7.4% of total) were tested for some IHC biomarker (ER, PR, HER2). The subtyping allowed doctors to adjust (56.7%) or confirm (43.3%) the initial therapeutic regimen. The average turnaround time was 56 days. Opportune IHC testing was found to be beneficial when it was available before or during the first rounds of chemotherapy.

**Conclusions:**

The use of data mining tools applied to health record data revealed that there is an association between timely immunohistochemistry and improved outcomes in breast cancer patients. Based on this finding, inclusion of turnaround time in clinical guidelines is recommended. As much of the health data in the country becomes digitized, our visualization tools allow a digital dashboard of the hospital service utilization histories.

**Electronic supplementary material:**

The online version of this article (10.1186/s12885-018-4833-4) contains supplementary material, which is available to authorized users.

## Background

In breast cancer, immunohistochemistry (IHC) is a critical part of the accepted standard of care for determining prognosis and response to therapy. A panel of three IHC markers—estrogen receptor (ER), progesterone receptor (PR), and human epidermal growth factor receptor 2 (HER2)—are the most commonly used due to its predictive value for chemotherapy response in breast cancer [1]. Despite the existence of other IHC markers (e.g. Ki67, p53) [2], and the development of several commercial multi-gene tests that improve prognosis and treatment selection [3], the availability and expense of these tests remain largely prohibitive for constrain-resourced hospital settings, regardless of country income.

Mexico is an emerging country with an upper-middle income economy (as considered by the World Bank), where the healthcare system still has challenges procuring enough resources. The Mexican consensus [4] and clinical practice guidelines for breast cancer [5–7], the main working documents for oncologists in the country, recommend routine use of a 3-marker panel (ER, PR, HER2) to guide management and treatment for patients. In Mexico, estimating the nationwide frequencies of molecular status is a challenging task due to the absence of cancer registries. However, two recent cohort studies in Mexico City [8, 9] showed that 56–64% of tumors had positive hormone receptor (ER, PR) status; 16–20% had HER2 positive status; and 23–26% had triple negative status. While these frequencies mirror those of other high and middle income countries, there is a troubling onset of disease among younger patients in Mexico (aged < 40), with a high prevalence of triple-negative breast cancers [10]. Furthermore, there is an increased mortality trend associated with breast cancer in the country [11]. Timely testing for these IHC biomarkers needs to be fully recognized in clinical guidelines and hospital policies, and the testing must account for the wide diversity of patient trajectories and hospital settings [12].

Recently, organizations that issue guidance on breast cancer care worldwide have been broadening their focus to include resource-stratified guidelines, which reflects the diversity of hospital settings in the world:

- The Breast Health Global Initiative (BHGI) guideline for breast cancer healthcare in low-income and middle-income countries [13] offers four levels of guidance: basic (hormone receptor status by empiric assessment or response to treatment); limited (determination of ER status by IHC); enhanced (determination of PR status by IHC and measurement of HER2 overexpression); and maximal (gene profiling testing).
- The National Comprehensive Cancer Network (NCCN) framework for oncology care resource-stratification [14] offers four levels of guidance equivalent to those in the BHGI guideline.
- The American Society for Clinical Oncology (ASCO) offers a resource-stratified guideline for cervical cancer [15], which can be used to illustrate the need for a similar guideline for breast cancer.
- The World Health Organization (WHO) [16] offers guidance to countries on three resource scenarios (low, medium, high) with differential actions in the national cancer control programs, but has not yet offered a breast cancer specific resource-stratified guideline.

In this study, we aimed to quantify the potential effect that timely IHC testing has in improving patient outcomes. We hypothesized that with the use of data mining on digitized hospital records it is possible to facilitate the quantification of IHC testing as well as other services offered to breast cancer patients. At the same time, this study provided hospital administrators with a visual monitoring tool that facilitates the burden of human-intensive labor to quantify critical procedures in resource-constrained hospitals (including IHC testing).

## Methods

### Hospital setting

The Mexican Institute of Social Security is a hybrid single-payer system with an integrated network of hospitals nationwide. In Michoacan (a state located in western Mexico), the General Regional Hospital No. 1 (HGR1) is the designated secondary-care facility for the IMSS-insured population, estimated at 1,288,695 people (28% of the state’s population), according to the 2015 census [17]; of these, 202,547 are women with ages between 40 to 69 years, which correspond to the target group for mammography screening for breast cancer. While there is a lot of heterogeneity between states in Mexico, it is important to highlight that the state of Michoacan ranks 29 out of 32 states in terms of competitiveness, governmental efficiency, and overall wealth [18].

The HGR1 hospital is located in a suburban community adjacent to Morelia (the capital of Michoacan). HGR1 is the secondary-care facility that serves as reference for seven General Zone Hospitals and 45 Family Medicine Units within the state of Michoacan. HGR1 has an oncology unit with a full complement of fixed staff and facilities available to all patients. It also offers pathology services, diagnostic imaging, and therapeutic capabilities with access to all approved drugs. Patients with breast cancer who require chemotherapy are sent to the outpatient medical unit, and patients with breast cancer who require radiation therapy are provided the service through an outsourced private service in the same city. The personnel include gynecologists, medical oncologists, surgical oncologists with significant breast cancer training, adequate numbers of nursing and pharmacy staff, surgeons with significant training, and cancer pathologists.

### Patients

The study population for this research was all cumulative breast cancer patients seen in 2014 by medical staff at HGR1, as reported by the institution’s breast cancer census in the absence of a cancer registry. To avoid selection bias in our study, we did not restrict the selection of patients to members of the female sex or any age group.

The general process at IMSS for breast cancer diagnosis is as follows. A patient with suspected breast cancer is referred from a primary-care facility to HGR1 after being examined by his/her family physician. If the physician suspects the existence of a breast lesion, the patient undergoes an imaging assessment using the breast imaging-reporting and data system (BIRADS) score. Upon arriving at HGR1, the patient is either seen by the breast cancer clinic (patients with BIRADS score 0 and 3) for further imaging studies and/or fine-needle biopsy assessments, or directly referred to the medical oncology service (patients with BIRADS score 4, 5 and 6), at which point they undergo surgical biopsies for pathology analysis. The gold-standard diagnosis of breast cancer is then provided by a board-certified pathologist, who assigns one of the C50 codes (malignant neoplasms of breast) from the international classification of diseases version 10 (ICD 10).

The final inclusion criteria were those whose biopsies had been tested with IHC to detect ER, PR, HER2 antibodies; then. We reviewed the medical records (both electronic and paper) of all breast cancer patients at HGR1, selected only those with IHC testing, and followed their medical histories, ending with March 2017, making note of multiple medical visits to the breast cancer clinic, medical oncology service, and/or surgical oncology service. From their medical records, we extracted information about IHC testing (antibody ordered, date of ordering, date of results being obtained), chemotherapy and hormonal drugs administered, and radiation sessions and surgical procedures undertaken.

### Study design

This was a retrospective hospital-based cohort study, using medical records collected routinely as part of clinical care. The objective was to understand the therapeutic impact on breast cancer patients of the time taken to test immunohistochemistry biomarkers in a resource-constrained hospital in western Mexico. We built a digital monitoring tool to report the frequency of treatment selection or treatment adjustment frequencies depending on breast cancer subtyping and the turnaround times for immunohistochemistry biomarker testing. The STROBE checklist is provided in Additional file 1. All analyses were performed in R version 3.3.2.

#### Patient trajectory monitoring

We explored the paper- and electronic-based medical records for all patients with IHC testing, from the patient’s first day at the hospital to the last follow-up pertaining to this study (occurring before March 2017). First, author MFRM manually reviewed the patient’s medical records and curated a set of possible events experienced by the patients of this study, annotating the time points for each event for each patient. Then, author ALP annotated those events with standardized coding from the Unified Medical Language System (UMLS). When disagreement occurred, both authors discussed the translation to assign a UMLS code to the medical records in Spanish. Finally, similar events were grouped in top-level categories. The UMLS codes, UMLS descriptions, Spanish description, and the top-level categories can be seen in Table 1.

**Table 1:** List of medical events with UMLS codes and descriptions in English and Spanish

UMLS code	UMLS description	Description in Spanish
Visit		
C0008952	Clinic Visits	*Atención inicial en la unidad de medicina familiar (primer nivel de atención).*
C2153644	Visit for: gynecological exam	*Atención inicial en la consulta de ginecología (segundo nivel de atención).*
Oncology		
C1620996	Oncology; primary focus of visit; work-up, evaluation, or staging at the time of cancer diagnosis or recurrence	*Atención inicial en la consulta de oncología médica.*
C1617848	Oncology; primary focus of visit; expectant management of patient with evidence of cancer for whom no cancer- directed therapy is being administered or arranged at present	*Atención subsecuente en la consulta de oncología médica.*
Request lab		
C2186763	Request lab results from pathology	*Solicitud de estudios de patología.*
C2186756	Request lab results from hematology	*Solicitud de estudios básicos de laboratorio clínico.*
C2186777	Request lab results from x-ray	*Solicitud de estudios de imagen (tele de tórax, ultrasonido, mastografía).*
C2186774	Request lab results from CT	*Realización de estudios especiales de imagen (tomografía).*
C2186775	Request lab results from MRI	*Realización de estudios especiales de imagen (resonancia magnética).*
Biopsy / Pathology		
C0177666	Needle biopsy of breast	*Realización de biopsia con aguja fina o gruesa.*
C0585992	Surgical biopsy of breast	*Realización de biopsia por escisión quirúrgica.*
C0807321	Pathology report	*Reporte de estudio histopatológico.*
Chemotherapy		
C0086965	Selection for Treatment	*Selección de tipo de tratamiento (quimioterapia, radioterapia, cirugía).*
C4302504	Chemotherapy started	*Inicio de quimioterapia.*
Surgery / Radiation		
C0436382	Radiotherapy started	*Inicio de radioterapia.*
C0024881	Mastectomy	*Realización de mastectomía radical.*
C0851238	Lumpectomy of breast	*Realización de lumpectomía*
IHC results		
C3248285	Quantitative HER2 immunohistochemistry (IHC) evaluation of breast cancer consistent with the scoring system defined in the ASCO/CAP guidelines (PATH)	*Solicitud de estudios de inmunohistoquímica (Receptor de HER2).*
C3248286	Quantitative non-HER2 immunohistochemistry (IHC) evaluation of breast cancer (eg, testing for estrogen or progesterone receptors [ER/PR]) performed (PATH)	*Solicitud de estudios de inmunohistoquímica (Receptores de estrógeno y progesterona).*
Treatment adjusted		
C1627778	Treatment adjusted per protocol	*Ajuste de tratamiento de primera línea (posterior al primer ciclo de quimioterapia).*
C0419989	Hormone replacement therapy started	*Inicio de tratamiento de reemplazo hormonal.*
Adverse events		
C0019993	Hospitalization	*Complicación de proceso oncológico (internamiento a causa de cáncer)*
C1282471	Local recurrence of malignant tumor of breast	*Recaída de proceso oncológico (mismo sitio tumor primario). Recidiva local de tumor maligno de mama.*
C3694291	Metastasis from malignant neoplasm of breast	*Reconocimiento de metástasis (tumor secundario).*
C13065577	Death (finding)	*Fallecimiento / Muerte*
Remission		
C0687702	Cancer Remission	*Evolución favorable de paciente, mejoría clínica; remisión.*

#### Missing data

Missing data was assumed to be non-missing at random (NMAR), and the events from Table 1 were considered missing for a specific reason. For example, if the code C2186775 (request lab result from MRI) was missing from a patient’s medical record, we left that value unassigned, since it was assumed that an MRI was not requested for that patient. There is a small chance that this assumption might be violated (and introduce some bias) if the physician did request the exam/procedure but forgot to write it down in the medical record. However, we did not impute any missing data, since that might be a larger source of bias for this EHR-based data.

#### Outliers

We assessed the time-to-event distribution for each event in Table 1, and reported the distribution of the patient cohort in violin plots that included: the median turnaround time, the interquartile probability density estimation, and the 95% confidence interval (whiskers). Outliers were identified using the 1.5 interquartile rule, but were not removed from the downstream analysis due to scarcity of data.

#### Clustering

We searched for groups of patients that had similar journeys in their hospital care. More technical details about the hierarchical clustering method are shown in Additional file 2. The input was a matrix of the number of days when events from Table 1 occurred for every patient, relative to their initial day of diagnosis in the hospital (day 0). Given that missing events were considered to be not missing at random (NMAR), they were filled with a large negative value (− 1000 for this cohort). Then, a patient similarity distance was calculated using Euclidean distance between these time-to-event values for every pair of patients. An agglomerative hierarchical clustering was constructed using complete linkage, the distance between the two furthest points in two clusters. The selection of the appropriate number of clusters was done through cluster stability analysis.

## Results

Our data mining efforts revealed the epidemiological information pertaining to this IMSS hospital as shown in Table 2. In this cohort, all patients were female. The highest prevalence of cancer was within the 50 to 59 year-old age group. There were 207 patients (51%) diagnosed with early stages of cancer (stages I and II). Only 30 patients (7%) had undergone estimated subtyping with IHC testing. Of all patients, 18 patients (4%) were younger than 40 years old. At this hospital, 23 patients (6%) had a BIRADS score of 2 or lower, meaning they should not have been sent to the secondary care facility. In these cases, patients had external biopsy investigations in non-IMSS hospitals (usually private) and decided to continue their care for follow-up treatment at the IMSS oncology services.

**Table 2:** Clinical characteristics of the cohort and receptor status for patients with IHC testing

Characteristic	Total (%)	With IHC testing (%)
Overall	*N* = 402	*N* = 30
Sex		
Female	402 (100%)	30 (100%)
Male	0	0
Age at diagnosis		
< 40	18 (4%)	3 (10%)
40–49	62 (15%)	16 (53%)
50–59	174 (43%)	7 (23%)
60–69	127 (32%)	4 (13%)
70+	21 (5%)	0
BIRADS		
0,1,2	23 (6%)	18 (60%)
3	203 (50%)	7 (23.3%)
4,5,6	162 (40%)	4 (13.3%)
unknown	14 (3%)	1 (3.3%)
Tumor stage		
I	85 (21%)	2 (7%)
II	122 (30%)	12 (40%)
III	84 (21%)	12 (40%)
IV	19 (5%)	3 (10%)
unknown	92 (23%)	1 (3%)
Patients without IHC testing	372 (93%)	0
Patients with IHC testing	30 (7%)	30 (100%)
ER testing		
positive	n.a.	17 (57%)
negative		11 (37%)
unknown		2 (7%)
PR testing		
positive	n.a.	16 (53%)
negative		12 (40%)
unknown		2 (7%)
HER2 testing		
positive	n.a.	5 (17%)
negative		23 (77%)
unknown		2 (7%)
Additional IHC testing		
Ki67, P53	n.a.	1 (3%)
unknown		29 (97%)
Subtype according to the Mexican Consensus [4]		
Luminal A	n.a.	14 (47%)
Luminal B		3 (10%)
Basal-like/Triple negative		9 (30%)
HER2		2 (7%)
not enough information to assign		2 (7%)

### Digital monitoring

**Fig. 1:**
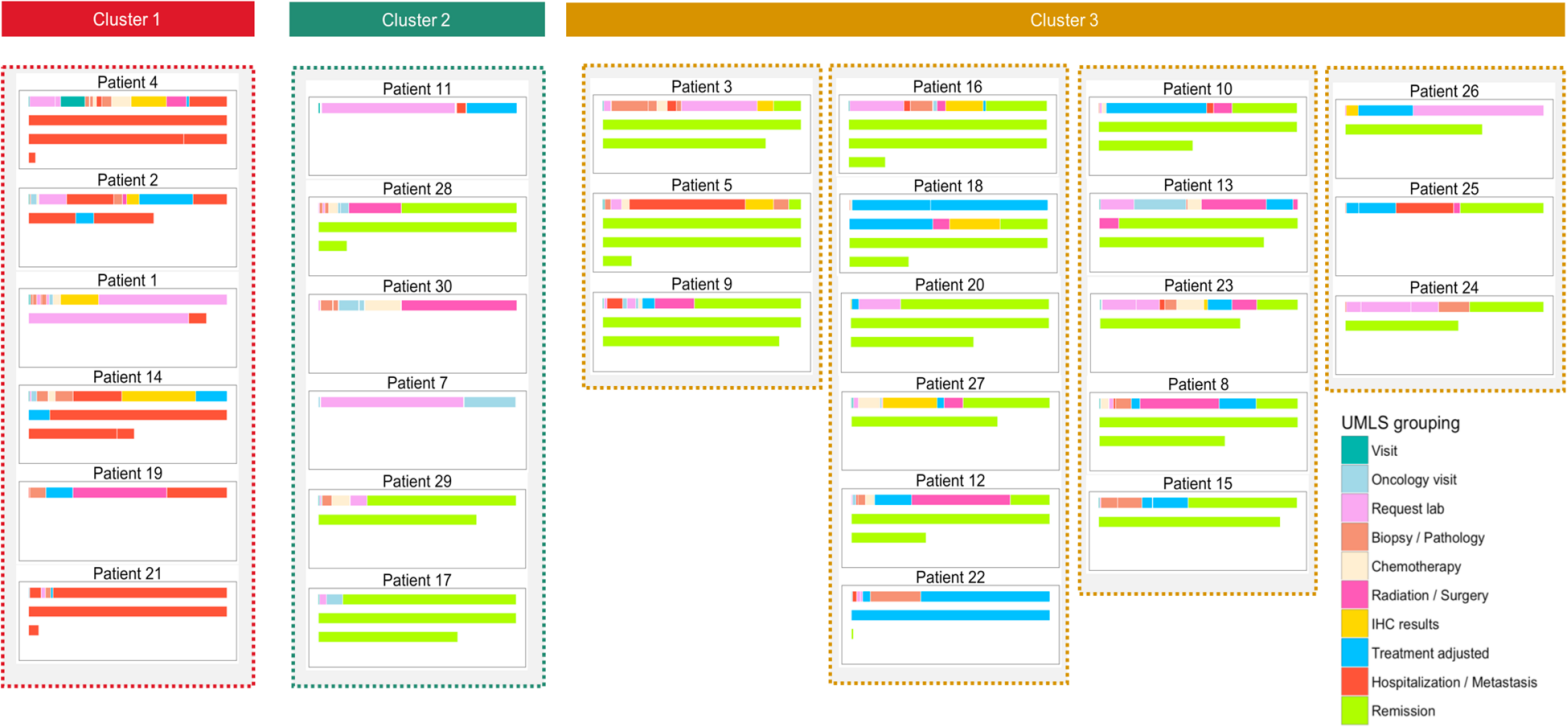
Medical trajectory plots of the cohort with IHC testing. Each patient is represented by a rectangle, with rows representing each year of follow-up treatment. Events are color-coded according to the type of event, and the length of the bar represents the duration between the occurrence of an event and the event that preceded it

Patient trajectories were finely segmented as shown in Fig. 1, which further allow digital analysis (and therefore optimization). Each patient is represented in a panel (rectangle) with colored bars, indicating the events that a patient experienced in the IMSS hospital. Each row represents one year of follow-up treatment for that patient, which can be from one to four rows (because the time maximum follow-up time span was three years and five months). The length of each colored bar represents the time between the occurrence of an event and the event that preceded it. Although events do not occur continuously, but, rather, happen at single time points (e.g. a patient visit, obtaining the results of a radiograph), the visual representation shown in Fig. 1 provides a sense of how much time was required for each event to occur, assuming nothing else happened at the same time.

### Turnaround time

**Fig. 2:**
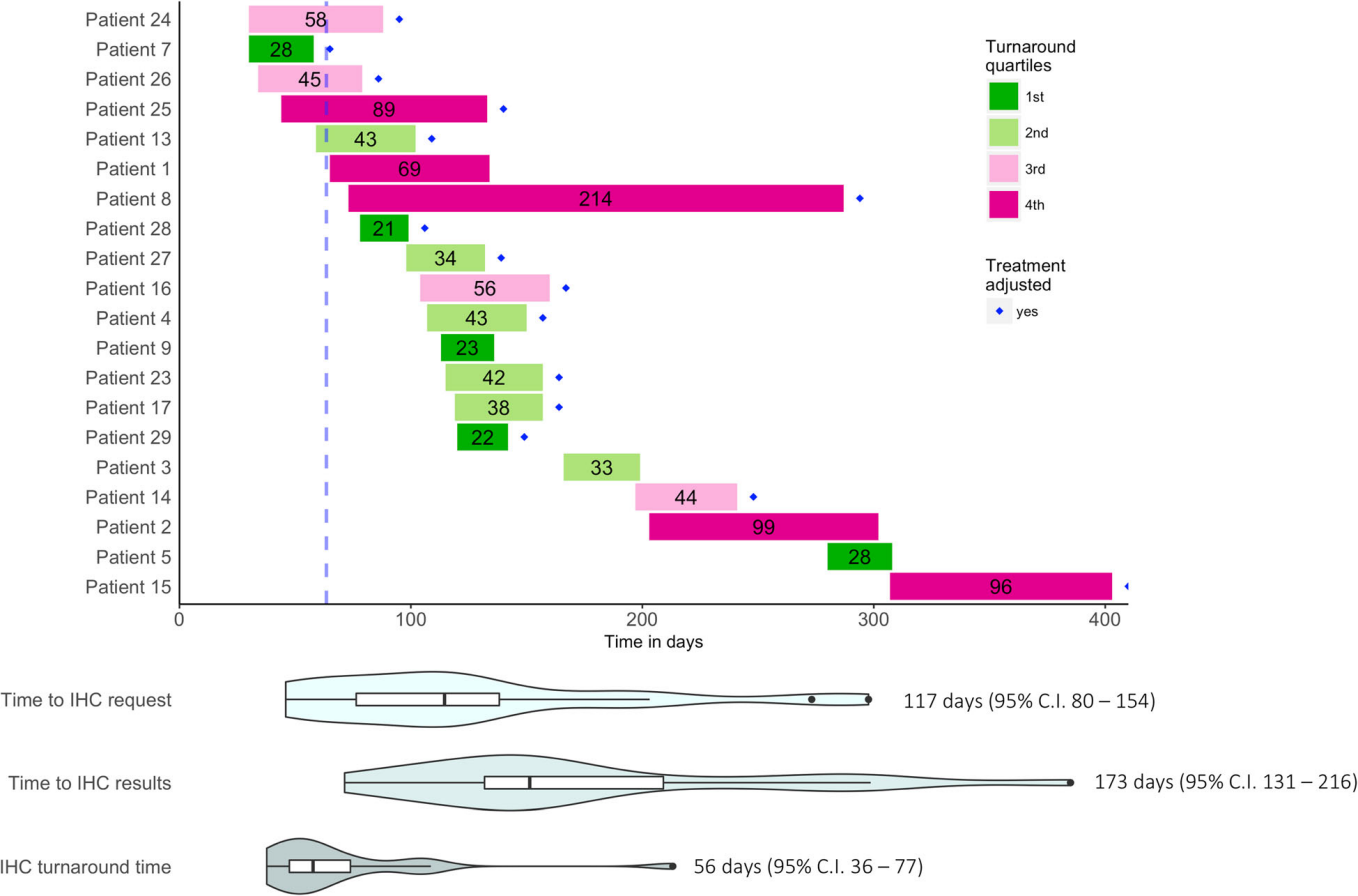
Turnaround times for IHC testing. The size of the bar indicates time in days from requesting results for IHC testing to obtaining them. For each patient, the initial time is the first day of follow-up treatment at the hospital. The color of the bar indicates the quartile within which the data falls in the distribution. The diamond mark indicates that the treatment was adjusted, according to protocols, after obtaining IHC results. The blue dashed line indicates the 1st quartile for the time it took before an IHC request was submitted. Violin plots with whiskers (equivalent to a boxplot with a probability density estimation) provide an overall view of the turnaround time needed for IHC testing in three categories: Time to IHC request, which represents the time from first day at hospital to request; Time to IHC result, which represent the time from first day at hospital to obtaining IHC results; IHC turnaround time, which represents the time from request of IHC to obtaining results

Further data mining on this cohort identified the turnaround time for IHC testing as shown in Fig. 2. Only 20 patients had information in their medical records relating to the timing of IHC testing. Although this service is referred to in the IMSS medical records, it is subcontracted to a private laboratory. There was large variation surrounding two key variables: a) the time needed to request a result, with an average time of 117 days (95% C.I. 80–154 days; and the turnaround time needed to obtain the results, with an average time of 56 days (95% C.I. 36–77 days). The total time to obtain a result from initial diagnosis was 173 days (95% C.I. 131–216 days). Overall, for these 30 patients with IHC testing, 17 patients (56.7%) had their treatments adjusted, and 13 patients (43.3%) had their treatments confirmed.

## Discussion

In Mexico, breast cancer continues to be a public health concern, even though more and more research is being done on its genomic changes and characteristics. Although our study investigated a small cohort of patients, the epidemiological characteristics among our cohort is similar to what has previously been estimated for the country (in regards to age group and staging) [11]. Our study further investigates the use of digital health mining tools for evaluating the timeliness of IHC testing, and the percentage of patients being tested. This tool can then be used to inform hospital administrators and public health officials.

### On the therapeutic value of IHC testing

Despite the small number of patients with IHC, the results suggest usefulness of the IHC in therapeutic decision-making and in determining the prognosis of the patient. In the hospital included in this study, therapeutic decisions are made based on clinical, surgical and histological results such as physical appearance, tumor size, lymphatic invasion, tumor necrosis, histological type, degree of differentiation and the number of affected lymph nodes. This study supports the therapeutic importance of immunophenotyping (the use of IHC testing for subtyping of cancer patients), as has been demonstrated broadly in other studies focusing on populations in high income settings. These results should be carefully considered and addressed in treatment plans created by the multidisciplinary care team. With the incorporation of information about receptor status, the traditional (FEC) chemotherapy regimen recommended by the IMSS protocol, composed by 5-fluorouracil, epirubicin, and cyclophosphamide, can be adjusted to improve treatment outcomes.

Patients with estrogen positive (ER+) and/or progesterone positive (PR+) could benefit from anti-hormone or endocrine therapy to prevent recurrence, either aromatase inhibitors to block estrogen production (e.g. anastrozole, letrozole, or exemestane), or the selective estrogen receptor modulators (SERMs), which interfere with the ability of estrogen to stimulate the growth of breast cells (e.g. tamoxifene or toremifene).

Additionally, patients with HER2 positive (HER2+), in which tumors tend to grow and spread more aggressively, could benefit from a targeted therapy that could block HER2 (e.g. trastuzumab or pertuzumab). The use of in situ hybridization (ISH), instead of IHC, can be used to determine HER2 status with an overall concordance with IHC, and it may be more beneficial to use both [19]. Furthermore, HER2 status has been successfully incorporated into medical practice to guide treatment decisions for breast cancer patients. In fact, the American Society of Clinical Oncology/College of American Pathologists (ASCO/CAP) updated their 2013 guidelines to designate more patients as eligible for trastuzumab therapy, in accordance with ISH and IHC testing [20]. Also, the BHGI resource stratified guidelines recommend the use of trastuzumab at the enhanced level due to the high cost of the drug, as well as for the availability for testing. The IMSS’s medications chart [21] reports trastuzumab as an available drug for any HER+ positive patient in the country.

### On the timeliness of IHC testing

Opportune IHC testing, understood as the availability of IHC results before the beginning of any treatment, is of critical importance. For the IMSS hospital in this study, IHC results were typically not timely opportune, but whenever they were available they triggered a response from the clinical oncology team to adjust patient treatments. The wide dispersion of time to request and turnaround time demonstrates the lack of standardization of this process. Many factors could have contributed to the delay in IHC testing, including: patient non-adherence to appointments; hospital overflow, resulting in delayed appointments; logistical problems between IMSS and the external laboratory service; and administrative problems within the hospital resulting in inadequate tracking of tests and results.

It is important to establish an appropriate timeline for opportune IHC testing. For example, In the United States, the College of American Pathologists’ (CAP) guideline on turnaround time for biopsy testing is around two days for 90% of cases [22]. In fact, the joint guideline on HER2 testing from the American Society of Clinical Oncology (ASCO) and CAP recommends informing the patients about the expected turnaround time [23]. In Australia, a study revealed that the average turnaround time was between 4 and 5 days [24]. In Saudi Arabia, a study showed that 24% of cases fall outside the recommended CAP turnaround time [25]. The European Society for Medical Oncology (ESMO) published a survey on 24 European countries were the turnaround time was 10 days or less for 89% of laboratories [26]. In the United Kingdom, the Royal College of Pathologists recommend the use of key performance indicators to have a histopathology diagnosis within seven days of biopsy in 90% of cases [27].

Given their resource-available settings, clinical guidelines in developed countries do not have a recommendation regarding the maximum turnaround time that could be effective in a patient’s treatment trajectory. More importantly, they do not address the time between initial diagnosis, molecular testing, and the selection of treatment. In Mexico, clinical guidelines for breast cancer recommend testing for ER, PR, and HER2 as part of the histopathology study, but fail to provide guidance regarding turnaround time. IMSS maintains a medical procedures manual, which includes ten key indicators for breast cancer screening, diagnosis, and treatment [28]. This manual measures time-to-diagnosis in a 30-day period, including imaging through mammography and results of histopathology report. In addition, the institution also measures time-to-treatment measured from the date of diagnosis, which should be achieved within a 21-day period. Recognizing the resource constraints of IMSS, the implementation of a key indicator policy related to IHC testing might significantly reduce turnaround times.

### On the need to monitor the percentage of patients being tested

The monitor shown in Fig. 1 quickly provides a patient history overview of hospital care, while Fig. 2 shows the detailed information for turnaround time. With more work on the user interface, we envision a tool that could eventually represent a valuable visual aid for hospital administrators, providing a quick overview of the patients seen in their hospital, which could be used as a decision-making tool, with the proper validation.

The low percentage (7.4%) of patients observed to be tested requires special attention. A common concern of retrospective chart review studies is underreporting. The amount of recorded information in the hospital included in our study can be improved by providing training to the clinical and administrative staff about the importance of requesting and reporting IHC testing. In the absence of a cancer registry, our best estimate of the disease is the institutional census, which does not record IHC testing results. As the hospitals in Mexico, and elsewhere, continue to become more and more electronic, there is a need to develop better software tools to facilitate recording and analyzing clinical information, including medical natural language processing and machine learning applications.

## Conclusions

Adverse events in the trajectory of an oncological patient, which might include hospitalization related to cancer, recurrence of tumor, or metastasis, are extremely costly for the healthcare system. The use of IHC testing was shown in our study to help with the selection of precise treatment for patients (either by adjusting the treatment or confirming it). The time in which IHC testing is performed is of critical importance if we want to influence improved prognosis.

We have revealed in this cohort that the use of opportune IHC testing is associated with beneficial therapeutic effects on breast cancer patients. The aims of any healthcare system should be the identification of earlier events that can have an impact on downstream events in the trajectory of an oncological patient’s treatment. In resource-constrained settings, it is important not only to consider alternatives to more costly diagnostics (e.g. genomic testing), but also to incorporate the regulatory and logistical aspects of implementing these tests.

IMSS must face the important challenge of continuing to improve their turnaround times, which should have a positive impact on the prognosis of their patients. As the Mexican healthcare system continues to transition from reactive to preventative care, the need for more IHC testing in breast cancer and other diseases will certainly allow for the further development of digital patient monitoring.

## Additional files

Additional file 1: STROBE Statement Checklist. (PDF 98 kb)

Additional file 2: Clustering analysis. (PDF 760 kb)

Additional file 3: Spanish translation. (PDF 675 kb)
