# Supplementary material for: Data mining of digitized health records in a resource-constrained setting reveals that timely immunophenotyping is associated with improved breast cancer outcomes
Source: BMC Cancer. 2018 Sep 27;18:933. doi: 10.1186/s12885-018-4833-4 (PMC6161369; doi:10.1186/s12885-018-4833-4)
Supplement: Supplementary file 1 — STROBE Statement Checklist. (PDF 98 kb) [file 12885_2018_4833_MOESM1_ESM.pdf]

Data mining of digitized health records in a resource-constrained setting reveals that timely immunophenotyping is associated with improved breast cancer outcomes

STROBE Statement—Checklist of items that should be included in reports of ***cohort studies***

	Item No	Recommendation	Author's assessment
**Title and abstract**	1	(*a*) Indicate the study's design with a commonly used term in the title or the abstract	See Methods in the Abstract.
		(*b*) Provide in the abstract an informative and balanced summary of what was done and what was found	See Methods and Findings in the Abstract.
**Introduction**			
Background/rationale	2	Explain the scientific background and rationale for the investigation being reported	See Introduction, paragraphs 1-3
Objectives	3	State specific objectives, including any prespecified hypotheses	See Introduction, last paragraph
**Methods**			
Study design	4	Present key elements of study design early in the paper	See Study Design in the Methods section.
Setting	5	Describe the setting, locations, and relevant dates, including periods of recruitment, exposure, follow-up, and data collection	See Hospital Setting in the Methods section
Participants	6	(*a*) Give the eligibility criteria, and the sources and methods of selection of participants. Describe methods of follow-up	See Patients in the Methods section paragraph 1.
		(*b*) For matched studies, give matching criteria and number of exposed and unexposed	Not applicable.
Variables	7	Clearly define all outcomes, exposures, predictors, potential confounders, and effect modifiers. Give diagnostic criteria, if applicable	See Patient Trajectory Monitoring, and Table 1 in Study Design of the Methods Section.
Data sources/ measurement	8*	For each variable of interest, give sources of data and details of methods of assessment (measurement). Describe comparability of assessment methods if there is more than one group	See Patients in the Methods section paragraph 1.
Bias	9	Describe any efforts to address potential sources of bias	See Patients in the Methods section, and Missing data in the Study Design Section paragraph 2.
Study size	10	Explain how the study size was arrived at	See Patients in the Methods section paragraph 1.

Quantitative variables	11	Explain how quantitative variables were handled in the analyses. If applicable, describe which groupings were chosen and why	See Patient Trajectory Monitoring, and Table 1 in Study Design of the Methods Section.
Statistical methods	12	(*a*) Describe all statistical methods, including those used to control for confounding	See Study Design of the Methods Section.
		(*b*) Describe any methods used to examine subgroups and interactions	See Clustering of the Study Design Subsection and Supplementary Material 2
		(*c*) Explain how missing data were addressed	See Missing data of the Study Design Subsection and Supplementary Material 2
		(*d*) If applicable, explain how loss to follow-up was addressed	Not applicable since it is a retrospective study, however, missing information was addressed in the limitations of the Discussion Section (On the need to monitor the percentage of patients being tested)
		(*e*) Describe any sensitivity analyses	Not applicable
**Results**			
Participants	13*	(a) Report numbers of individuals at each stage of study—e.g. numbers potentially eligible, examined for eligibility, confirmed eligible, included in the study, completing follow-up, and analysed	See Results, paragraph 1; and Table 2.
		(b) Give reasons for non-participation at each stage	See Results, paragraph 1.
		(c) Consider use of a flow diagram	A flow diagram would be too simple, since inclusion criteria is only one variable (IHC testing or not). Table 2 is sufficient.
Descriptive data	14*	(a) Give characteristics of study participants (e.g. demographic, clinical, social) and information on exposures and potential confounders	See Table 2.
		(b) Indicate number of participants with missing data for each variable of interest	See Table 2.
		(c) Summarise follow-up time (eg, average and total amount)	See Figure 1 for details of follow-up for each patient.
Outcome data	15*	Report numbers of outcome events or summary measures over time	See Figure 1 for details of outcomes for each patient.
Main results	16	(*a*) Give unadjusted estimates and, if applicable, confounder-adjusted estimates and their precision (eg, 95% confidence interval). Make clear which confounders were adjusted for and why they were included	See Figure 2.

		(*b*) Report category boundaries when continuous variables were categorized	Not applicable.
		(*c*) If relevant, consider translating estimates of relative risk into absolute risk for a meaningful time period	Not applicable.
Other analyses	17	Report other analyses done—e.g. analyses of subgroups and interactions, and sensitivity analyses	Not applicable.
**Discussion**			
Key results	18	Summarise key results with reference to study objectives	See first paragraph in the Discussion section
Limitations	19	Discuss limitations of the study, taking into account sources of potential bias or imprecision. Discuss both direction and magnitude of any potential bias	See "On the need to monitor the percentage of patients being tested" in the Discussion section
Interpretation	20	Give a cautious overall interpretation of results considering objectives, limitations, multiplicity of analyses, results from similar studies, and other relevant evidence	See "On the timeliness of IHC testing" in the Discussion section
Generalisability	21	Discuss the generalisability (external validity) of the study results	See "On the therapeutic value of IHC testing" in the Discussion section
**Other information**			
Funding	22	Give the source of funding and the role of the funders for the present study and, if applicable, for the original study on which the present article is based	See Funding in the Declarations section

*Give information separately for exposed and unexposed groups.

Note: An Explanation and Elaboration article discusses each checklist item and gives methodological background and published examples of transparent reporting. The STROBE checklist is best used in conjunction with this article (freely available on the Web sites of PLoS Medicine at http://www.plosmedicine.org/, Annals of Internal Medicine at http://www.annals.org/, and Epidemiology at http://www.epidem.com/). Information on the STROBE Initiative is available at http://www.strobe-statement.org.
