# Supplementary material for: Data mining of digitized health records in a resource-constrained setting reveals that timely immunophenotyping is associated with improved breast cancer outcomes
Source: BMC Cancer. 2018 Sep 27;18:933. doi: 10.1186/s12885-018-4833-4 (PMC6161369; doi:10.1186/s12885-018-4833-4)
Supplement: Supplementary file 2 — Clustering analysis. (PDF 760 kb) [file 12885_2018_4833_MOESM2_ESM.pdf]

Data mining of digitized health records in a resource-constrained setting reveals that timely immunophenotyping is associated with improved breast cancer outcomes

Arturo López-Pineda, PhD, Mario F. Rodríguez-Moran, MD, Cleto Álvarez-Aguilar, MD, MS, Sarah M. Fuentes Valle, MD, Román Acosta-Rosales, MD, Ami S. Bhatt, MD, PhD, Shruti N. Sheth, MD, Carlos D. Bustamante, PhD

Hierarchical Clustering

Clustering is an unsupervised machine learning task to classify samples of a population into homogeneous subsets, or clusters, which correspond to subpopulation structures in a dataset[1]. Hierarchical clustering is a popular technique that creates clusters with a predetermined ordering (hierarchy), and we selected the agglomerative (bottom-up) algorithm. This method, starts by assigning each sample to its own cluster. Then, it computes a similarity score (distance) between each of the pairwise samples, and join the two most similar ones into a larger cluster. The algorithm continues to recursively aggregate clusters until all samples have been added to the hierarchy.

Hierarchical clustering is dependent of being able to calculate distance matrix between each sample, given a distance function. The matrix gets continuously updated to display distances between clusters. The hierarchical clustering can be visualized using a dendrogram, a highly informative description of the clustering as a binary tree structure. Figure 1 shows the steps that we took from recording time-to-event data, to creating distance a matrix of pairwise samples, to creating a hierarchical clustering dendrogram.

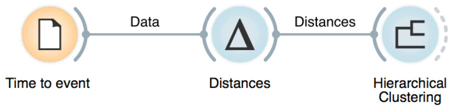

Figure 1. Diagram representing steps needed from data to clustering

Pairwise distance scoring (*d*). We selected an Euclidean distance, which calculates the square root of the sum of the square difference between two vectors (x_1, x_2). The vectors represent the time-to-event matrix collected from the medical records. The main manuscript shows a list of variables (in Table 1) that were recorded, and how many days passed for that event to occur, relative to the first day at the hospital (day 0). The order in which events are recorded in time-to-event matrix does not affect the calculation of the Euclidean distance, given that the pairwise difference at each event is squared independently, and then summed up (see Equations 1).

$$d(x_1, x_2) = \sqrt{\sum_{i=1}^{n} (f_i - g_i)^2}$$ Eq. 1

Figure 2 shows an example of two samples (Patient 1 and 2), with its corresponding time-to-event feature vector in days. The pairwise distance calculation between these two patients, using the Euclidean distance, can be seen in Equations 2 and 3. The distance (d) between patient 1 ($p1$) and patient 2 ($p2$) is computed by having the squared difference between each feature list of patient 1 (f_i) and the features for patient 2 (g_i).

$f_i = feaures\ of\ Patient\ 1$

Patient 1	1	3	7	44	37	21	14	659	?	7	14	32	58	58	?	23	?	128	128	692	692	23	692	692
Patient 2	1	3	14	18	3	17	156	69	?	3	156	172	17	17	180	156	?	203	203	302	484	156	451	595

$g_i = feaures\ of\ Patient\ 2$

Figure 2. Day-to-event matrix example

$$d(p1, p2) = \sqrt{(f_1 - g_1)^2 + (f_2 - g_2)^2 + \ldots + (f_n - g_n)^2}$$ Eq. 2

$$d(p1, p2) = \sqrt{(1 - 1)^2 + (3 - 3)^2 + \ldots + (692 - 595)^2}$$ Eq. 3

In the previous calculation, there is a need to include values in the feature space. Our recorded data has missing values which we assumed to be missing not at random (MNAR). For example, a given patient might have had a lumpectomy done, while another patient might have had a mastectomy (or both). If the information was missing from the record, we assumed that the reason that it was missing was not at random. Instead, the missing information was missing because the patient did not have the procedure done. Typically, the way to deal with this MNAR property is to assign a missing value or missing category that represents the fact that the element is missing. In our case, we selected a large negative number given our day-to-event values, in our case -1,000.

We did not consider the missing values to be missing at random (MAR) or missing completely at random (MCAR) because of the nature of the procedures. Typically, the way to deal with those missing values would have been to delete rows or columns from the analysis (in our case deleting the entire matrix); performing a partial analysis (subsampling); replacing missing values with means or other imputation methods. However, these interpretations would have introduced additional error to our cohort analysis.

Linkage (*L*) distance between clusters (*r, s*). We selected the complete linkage method, which calculates the distance (D) between the two furthest points in both clusters, as seen in Equation 4. Other popular linkage methods that could be used include single (distance between closest points), average (average distance between all points in both clusters); centroid, etc. We did not investigated these different methods.

$$Complete\ Linkage(r, s) = \max\left(D\left(x_{ri}, x_{sj}\right)\right) \quad \text{Eq. 4}$$

Figure 3 shows the time in which each event occurred for each patient is measured using information found in their medical record (in days, positive). To be able to create a dissimilarity matrix to calculate distance between patients, all missing values were assigned a negative value of -1,000. Three main groups of patients (or clusters) were identified in this graph. This figure does not provide a context for the ordering in which events occurred, but it can still help to provide information about patients with similar trajectories. The dendrograms was created following the hierarchical clustering approach described earlier.

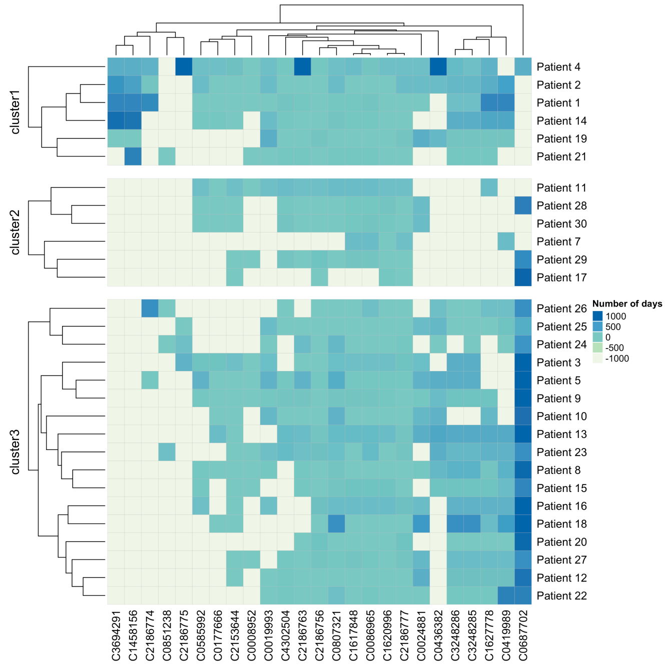

Figure 3. Heatmap of time-to-event with patient clustering.

<u>Technology</u>. We used the R programming language version 3.3.2 under a 64-bit Unix platform (Apple®). Our visualization monitoring tool can be downloaded from our Github repository (https://github.com/bustamante-lab/patientJourney). The script that we used for the cluster analysis used the package 'ComplexHeatmap'[2] version 1.18.1 from the Bioconductor repository release 3.7, as shown in Code 1.

Code 1

```
Heatmap(y,
   name ="Number of days",
   col = colorRamp2(c(-1000, -500, 0, 500, 1000),
      c("#f0f9e8", "#bae4bc", "#7bccc4", "#43a2ca", "#0868ac")),
   km = 3,
   clustering_distance_rows = "euclidean",
   clustering_method_rows = "complete",
   clustering_distance_columns = "euclidean",
   clustering_method_columns = "complete",
   row_dend_width = unit(3, "cm"),
   column_dend_height = unit(2, "cm"),
   rect_gp = gpar(col = "gray", lty = 1, lwd = 0.2),
   gap = unit(5, "mm")
)
```

Bibliography

1 Kimes PK, Liu Y, Neil Hayes D, Marron JS. Statistical significance for hierarchical clustering. *Biometrics* 2017; **73**: 811–21.

2 Gu Z, Eils R, Schlesner M. Complex heatmaps reveal patterns and correlations in multidimensional genomic data. *Bioinformatics* 2016; **32**: 2847–9.
