# Supplementary material for: Data mining of digitized health records in a resource-constrained setting reveals that timely immunophenotyping is associated with improved breast cancer outcomes
Source: BMC Cancer. 2018 Sep 27;18:933. doi: 10.1186/s12885-018-4833-4 (PMC6161369; doi:10.1186/s12885-018-4833-4)
Supplement: Supplementary file 3 — Spanish translation. (PDF 675 kb) [file 12885_2018_4833_MOESM3_ESM.pdf]

La minería de datos de expedientes clínicos digitalizados, en un entorno de recursos limitados, revela que la inmunofenotipificación oportuna se asocia con mejor resultados en cáncer de mama

Arturo López-Pineda, PhD, Mario F. Rodríguez-Moran, MD, Cleto Álvarez-Aguilar, MD, MS, Sarah M. Fuentes Valle, MD, Román Acosta-Rosales, MD, Ami S. Bhatt, MD, PhD, Shruti N. Sheth, MD, Carlos D. Bustamante, PhD

[1] Departamento de Ciencia de Datos Biomédicos, Escuela de Medicina, Universidad Stanford, Stanford, California, EE.UU.

[2] Facultad de Ciencias Médicas y Biológicas "Dr. Ignacio Chávez", Universidad Michoacana de San Nicolás de Hidalgo. Morelia, Michoacán, México.

[3] Coordinación de Investigación en Salud, Delegación Michoacán, Instituto Mexicano del Seguro Social. Morelia, Michoacán, México.

[4] Servicio de Ginecobstetricia del Hospital General Regional No. 1, Instituto Mexicano del Seguro Social. Morelia Michoacán México.

[5] Delegación Michoacán, Instituto Mexicano del Seguro Social. Morelia Michoacán México.

[6] Departamento de Genética, Escuela de Medicina, Universidad Stanford, Stanford, California, EE.UU.

[7] Departamento de Medicina (Hematología), Escuela de Medicina, Universidad Stanford, Stanford, California, EE.UU.

[8] Departamento de Medicina (Oncología), Escuela de Medicina, Universidad Stanford, Stanford, California, EE.UU.

[§] Estos autores contribuyeron de forma equivalente a este trabajo.

[*] Autor para correspondencia

Enviar correspondencia a:

Arturo López Pineda, PhD
Departamento de Ciencia de Datos Biomédicos, Escuela de Medicina, Universidad Stanford
1265 Welch Road, MSOB X313, 94305, Stanford, California, Estados Unidos de América
Email: arturolp@stanford.edu

Resumen

Antecedentes. Las organizaciones que emiten guías sobre cáncer de mama recomiendan el uso de inmunohistoquímica (IHQ) para proporcionar una atención adecuada y precisa a los pacientes. Sin embargo, dada la diversidad de entornos hospitalarios en el mundo es necesaria la identificación de tiempos de respuesta máximos permisibles para las pruebas de IHQ. Muchos menos esfuerzos se han dedicado al desarrollo de herramientas digitales que permitan a los administradores de hospitales controlar el historial de utilización de los servicios que se ofrecen a sus pacientes.

Métodos. En este estudio retrospectivo de cohorte hospitalaria, revisamos los registros clínicos electrónicos y en papel de todos los pacientes con sospecha de cáncer de mama atendidos en un hospital de atención secundaria del Instituto Mexicano del Seguro Social (IMSS), ubicado en el occidente de México. Posteriormente, revisamos tres años de historial médico de los pacientes con pruebas de IHQ.

Resultados. En 2014, hubo 402 pacientes con cáncer de mama, de los cuales 30 (7.4 % del total) fueron evaluados con algún biomarcador de IHQ (RE, RP, HER2). El subtipo de cáncer permitió a los médicos ajustar (56·7%) o confirmar (43·3%) el régimen terapéutico inicial. El tiempo promedio de respuesta fue de 56 días. Se encontró que las pruebas oportunas de IHQ eran beneficiosas cuando estaban disponibles antes o durante las primeras rondas de quimioterapia.

Conclusiones. El uso de herramientas de minería de datos aplicadas a los expedientes clínicos reveló que existe una asociación entre las pruebas de inmunohistoquímica aplicadas de forma oportuna y mejores resultados para los pacientes con cáncer de mama. Sobre la base de este hallazgo, se recomienda incluir el tiempo de respuesta en las guías de práctica clínica. A medida que gran parte de los datos de salud en el país se digitalizan, nuestras herramientas de visualización permiten un panel digita" del historial de utilización de los servicios hospitalarios.

Palabras clave: Neoplasmas de la mama; Inmunohistoquímica; Biomarcadores tumorales; Registros médicos; Codificación clínica; Minería de datos; México.

1. Antecedentes

En cáncer de mama, la inmunohistoquímica (IHQ) es una parte fundamental del estándar de atención aceptado para determinar el pronóstico y la respuesta al tratamiento. Un panel de tres marcadores de IHQ: receptor de estrógeno (RE), receptor de progesterona (RP) y receptor 2 del factor de crecimiento epidérmico humano (HER2), son los más utilizados debido a su valor predictivo de la respuesta de la quimioterapia en cáncer de mama [1]. A pesar de la existencia de otros marcadores de IHQ (p. ej. Ki67, p53) [2] y el desarrollo de varias pruebas genéticas comerciales que mejoran el pronóstico y la selección del tratamiento [3], la disponibilidad y el gasto de estas pruebas siguen siendo prohibitivas para entornos hospitalarios con restricciones de recursos, independientemente de los ingresos del país.

México es un país emergente con una economía de ingreso medio-alto (según lo considerado por el Banco Mundial), donde el sistema de atención de la salud todavía tiene desafíos para conseguir recursos suficientes. El consenso mexicano [4] y las guías de práctica clínica para cáncer de mama [5-7], los principales documentos de trabajo para oncólogos en el país recomiendan el uso rutinario de un panel de 3 marcadores (RE, RP, HER2) para guiar el manejo y tratamiento de los pacientes. En México, debido a la ausencia de registros de cáncer, es un reto estimar las frecuencias nacionales del estado molecular. Sin embargo, dos estudios recientes de cohortes en la Ciudad de México [8,9] mostraron que el 56-64% de los tumores tenían un estado positivo de receptores hormonales (ER, PR); 16-20% tenían HER2 estado positivo; y 23-26% tenían estado triple negativo. Si bien estas frecuencias se asemejan a las de otros países de ingresos altos y medios, existe una aparición preocupante de la enfermedad entre los pacientes más jóvenes en México (<40 años), con una alta prevalencia de cánceres de mama triple negativos [10]. Además, existe una mayor tendencia de mortalidad asociada con el cáncer de mama en el país [11]. Las pruebas oportunas para estos biomarcadores de IHQ deben ser plenamente reconocidas en las directrices clínicas y las políticas hospitalarias, y las pruebas deben tener en cuenta la amplia diversidad de trayectorias de los pacientes y los entornos hospitalarios [12].

Recientemente, las organizaciones que emiten directrices sobre la atención del cáncer de mama en todo el mundo han ampliado su enfoque para incluir guías de práctica clínica estratificadas por disponibilidad de recursos, lo cual refleja la diversidad de entornos hospitalarios en el mundo:

- La guía para el cuidado de la salud del cáncer de mama en países de bajos y medianos ingresos de la Iniciativa Global de Salud Materna (BHGI, por sus siglas en inglés) [13] ofrece cuatro niveles de orientación: básico (estado de los receptores hormonales mediante evaluación empírica o respuesta al tratamiento); limitado (determinación del estado del RE utilizando IHQ); mejorado (determinación del estado del RP por IHQ y medición de la sobreexpresión de HER2); y máxima (prueba de perfiles genéticos).
- El marco de atención oncológica estratificado por recursos de la Red Nacional Integral del Cáncer (NCCN, en inglés) [14] ofrece cuatro niveles de orientación equivalentes a los de la guía BHGI.

- La Sociedad Estadounidense de Oncología Clínica (ASCO, en inglés) ofrece una guía estratificada de recursos para el cáncer de cuello uterino [15], que se puede utilizar para ilustrar la necesidad de una pauta similar para el cáncer de mama.
- La Organización Mundial de la Salud (OMS) [16] ofrece orientación a países sobre tres escenarios de recursos (bajo, medio, alto) con acciones diferenciales en los programas nacionales de control del cáncer, pero aún no ha ofrecido una directriz estratificada de recursos específicos para el cáncer de mama.

En este estudio, nuestro objetivo fue cuantificar el efecto potencial que las pruebas de IHQ oportunas tienen para mejorar los resultados de los pacientes. Nuestra hipótesis fue que con el uso de la minería de datos en los registros hospitalarios digitalizados es posible facilitar la cuantificación de las pruebas de IHQ, así como otros servicios que se ofrecen a los pacientes con cáncer de mama. Al mismo tiempo, este estudio proporcionó a los administradores del hospital una herramienta de monitoreo visual que facilita la carga de mano de obra intensiva en humanos para cuantificar los procedimientos críticos en los hospitales con recursos limitados (incluidas las pruebas de IHQ).

2. Métodos

Entorno hospitalario

El Instituto Mexicano del Seguro Social es un sistema híbrido de administrador único con una red integrada de hospitales en todo el país. En Michoacán (un estado ubicado en el occidente de México), el Hospital Regional General No. 1 (HGR1) es el hospital de atención secundaria designada para la población asegurada por el IMSS, estimada en 1,288,695 personas (28% de la población del estado), según el censo de 2015 [17]; de estos, 202,547 son mujeres con edades comprendidas entre 40 y 69 años, que corresponden al grupo objetivo para la detección de cáncer de mama por mamografía. Si bien hay mucha heterogeneidad entre los estados en México, es importante destacar que el estado de Michoacán ocupa el lugar 29 de 32 estados en términos de competitividad, eficiencia gubernamental y riqueza en general [18].

El hospital HGR1 está ubicado en una comunidad suburbana adyacente a Morelia (la capital de Michoacán). El HGR1 es la instalación de atención secundaria que sirve como referencia para siete hospitales generales de zona y 45 unidades de medicina familiar dentro del estado de Michoacán. El HGR1 tiene una unidad de oncología con una plantilla completa de personal fijo e instalaciones disponibles para todos los pacientes. También ofrece servicios de patología, diagnóstico por imágenes y capacidades terapéuticas con acceso a todos los medicamentos aprobados. Los pacientes con cáncer de mama que requieren quimioterapia se envían a la unidad médica ambulatoria, y los pacientes con cáncer de mama que requieren radioterapia reciben el servicio a través de un servicio privado subcontratado en la misma ciudad. El personal incluye ginecólogos, oncólogos médicos, oncólogos quirúrgicos con entrenamiento significativo

en cáncer de mama, un número adecuado de personal de enfermería y farmacia, cirujanos con entrenamiento significativo y patólogos especializados en cáncer.

Pacientes

La población de estudio para esta investigación fueron todos los pacientes acumuladas de cáncer de mama atendidas en 2014 por personal médico en el HGR1, según lo informado por el censo de cáncer de mama de la institución en ausencia de un registro de cáncer. Para evitar el sesgo de selección en nuestro estudio, no restringimos la selección de pacientes a miembros del sexo femenino o cualquier grupo de edad.

En el IMSS el proceso general para el diagnóstico de cáncer de mama es el siguiente: Un paciente con sospecha de cáncer de mama es derivado de un centro de atención primaria al HGR1 después de ser examinado por su médico familiar. Si el médico sospecha la existencia de una lesión mamaria, el paciente se somete a un estudio de imagen mediante el puntaje del sistema de información y obtención de imágenes de la mama (BIRADS, en inglés). Al llegar a HGR1, la clínica de mama (pacientes con puntaje BIRADS 0 y 3) ve al paciente para obtener más estudios de imagen y / o biopsias con aguja fina, o directamente al servicio de oncología médica (pacientes con puntaje BIRADS 4, 5 y 6), en cuyo caso se someten a biopsias quirúrgicas para el análisis de la patología. El estándar de oro para el diagnóstico de cáncer de mama es proporcionado por un patólogo, que asigna uno de los códigos C50 (neoplasias malignas de la mama) de la clasificación internacional de enfermedades versión 10 (CIE 10).

Los criterios de inclusión finales fueron aquellos cuyas biopsias habían tenido una prueba de IHQ para detectar anticuerpos de RE, RP, y HER2. Se revisaron los expedientes clínicos (electrónicos y en papel) de todos los pacientes con cáncer de mama en el HGR1, para seleccionar solo aquellos con pruebas de IHQ y seguir sus historias clínicas hasta marzo de 2017, tomando nota de las múltiples visitas médicas a la clínica de mama, servicio de oncología médica y/o servicio de oncología quirúrgica. A partir de sus expedientes clínicos, se extrajo información sobre las pruebas de IHQ (anticuerpos solicitados, fecha de solicitud, fecha de obtención de resultados), quimioterapia y tratamientos hormonales administrados, así como sesiones de radiación y procedimientos quirúrgicos realizados.

Diseño del estudio

Se trata de un estudio retrospectivo de cohorte hospitalaria que utiliza expedientes clínicos generados de forma rutinaria como parte de la atención clínica. El objetivo fue comprender si el tiempo que toma probar biomarcadores de inmunohistoquímica tiene un impacto terapéutico en los pacientes con cáncer de mama en un hospital con recursos limitados en el occidente de México. Creamos una herramienta de monitoreo digital para informar la frecuencia de la selección del tratamiento o las frecuencias de ajuste del tratamiento dependiendo del subtipo de cáncer de mama y los tiempos de respuesta para las pruebas de biomarcadores de inmunohistoquímica. La lista de verificación STROBE se proporciona en el Archivo Adicional 1. Todos los análisis se realizaron en R versión 3.3.2.

Monitoreo de la trayectoria del paciente. Exploramos los expedientes clínicos médicos en papel y electrónicos para todos los pacientes con pruebas de IHQ, desde el primer día del paciente en el hospital hasta el último seguimiento relacionado por este estudio (que tuvo lugar antes de marzo de 2017). Primero el autor MFRM revisó manualmente los registros médicos del paciente y seleccionó un conjunto de posibles eventos experimentados por los pacientes de este estudio, anotando los puntos de tiempo para cada evento para cada paciente. Luego, el autor ALP anotó esos eventos con codificación estandarizada del sistema de lenguaje médico unificado (UMLS, en inglés). Cuando hubo desacuerdo, ambos autores discutieron la traducción para asignar un código UMLS a los expedientes clínicos en español. Finalmente, los eventos similares fueron agruparon en categorías de alto nivel. En la Tabla 1 se pueden ver los códigos UMLS, sus descripciones UMLS en inglés, y la descripción en español, así como las categoerías de alto nivel utilizadas.

Tabla 1. Lista de eventos médicos con códigos UMLS y descripciones en inglés y español.

Código UMLS	Descripción UMLS en inglés	Descripción en español
**Visitas**		
C0008952	*Clinic Visits*	Atención inicial en la unidad de medicina familiar (primer nivel de atención).
C2153644	*Visit for: gynecological exam*	Atención inicial en la consulta de ginecología (segundo nivel de atención).
**Oncología**		
C1620996	*Oncology; primary focus of visit; work-up, evaluation, or staging at the time of cancer diagnosis or recurrence*	Atención inicial en la consulta de oncología médica.
C1617848	*Oncology; primary focus of visit; expectant management of patient with evidence of cancer for whom no cancer- directed therapy is being administered or arranged at present; cancer-directed therapy might be considered in the future*	Atención subsecuente en la consulta de oncología médica.
**Solicitud de estudios de laboratorio**		
C2186763	*Request lab results from pathology*	Solicitud de estudios de patología.
C2186756	*Request lab results from hematology*	Solicitud de estudios básicos de laboratorio clínico.
C2186777	*Request lab results from x-ray*	Solicitud de estudios de imagen (tele de tórax, ultrasonido, mastografía).
C2186774	*Request lab results from CT*	Realización de estudios especiales de imagen (tomografía).
C2186775	*Request lab results from MRI*	Realización de estudios especiales de imagen (resonancia magnética).
**Biopsia / Patología**		
C0177666	*Needle biopsy of breast*	Realización de biopsia con aguja fina o gruesa.
C0585992	*Surgical biopsy of breast*	Realización de biopsia por escisión quirúrgica.
C0807321	*Pathology report*	Reporte de estudio histopatológico.
**Quimioterapia**		
C0086965	*Selection for Treatment*	Selección de tipo de tratamiento (quimioterapia, radioterapia, cirugía).
C4302504	*Chemotherapy started*	Inicio de quimioterapia.
**Cirugía / Radiación**		
C0436382	*Radiotherapy started*	Inicio de radioterapia.
C0024881	*Mastectomy*	Realización de mastectomía radical.
C0851238	*Lumpectomy of breast*	Realización de lumpectomía
**Resultados de IHQ**		
C3248285	*Quantitative HER2 immunohistochemistry (IHC) evaluation of breast cancer consistent with the scoring system defined in the ASCO/CAP guidelines (PATH)*	Solicitud de estudios de inmunohistoquímica (Receptor de HER2).

C3248286	*Quantitative non-HER2 immunohistochemistry (IHC) evaluation of breast cancer (eg, testing for estrogen or progesterone receptors [ER/PR]) performed (PATH)*	Solicitud de estudios de inmunohistoquímica (Receptores de estrógeno y progesterona).
**Ajuste de tratamiento**		
C1627778	*Treatment adjusted per protocol*	Ajuste de tratamiento de primera línea (posterior al primer ciclo de quimioterapia).
C0419989	*Hormone replacement therapy started*	Inicio de tratamiento de reemplazo hormonal.
**Eventos adversos**		
C0019993	*Hospitalization*	Complicación de proceso oncológico (internamiento a causa de cáncer)
C1282471	*Local recurrence of malignant tumor of breast*	Recaída de proceso oncológico (mismo sitio tumor primario).
C3694291	*Metastasis from malignant neoplasm of breast*	Reconocimiento de metástasis (tumor secundario).
C13065577	*Death (finding)*	Fallecimiento / Muerte
**Remisión**		
C0687702	*Cancer Remission*	Evolución favorable de paciente, mejoría clínica; remisión.

Datos faltantes. Se asumió que los datos faltantes no se debían al azar (NMAR, en inglés), y los eventos de la Tabla 1 se consideraron faltantes por una razón específica. Por ejemplo, si el código C2186775 (solicitud de resultado de laboratorio de resonancia magnética) faltaba en la historia clínica de un paciente, dejamos ese valor sin asignar, ya que se asumió que no se solicitó una resonancia magnética para ese paciente. Hay una pequeña posibilidad de que esta suposición pueda no ser cierta (e introducir algún sesgo) si el médico solicitó el examen / procedimiento, pero se olvidó de anotarlo en el registro médico. Sin embargo, no imputamos ningún dato faltante, ya que podría ser una mayor fuente de sesgo para estos datos basados en expedientes clínicos electrónicos.

Valores atípicos. Se evaluó la distribución del tiempo hasta el evento para cada evento en la Tabla 1, y se informó sobre la distribución de la cohorte de pacientes en diagrama de violín que incluyeron: el tiempo medio de respuesta, la estimación de la densidad de probabilidad intercuartílica y el intervalo de confianza del 95% (bigotes). Los valores atípicos se identificaron utilizando la regla de 1.5 intercuantiles, pero no se eliminaron del análisis posterior debido a la escasez de datos.

Agrupamiento. Buscamos grupos de pacientes (clústeres) que tuvieron viajes similares en la atención hospitalaria. Más detalles técnicos sobre el método de agrupación jerárquica se muestran en el Archivo Adicional 2. La entrada fue una matriz del número de días en que ocurrieron los eventos de la Tabla 1 para cada paciente, en relación con el día inicial de diagnóstico en el hospital (día 0). Dado que los eventos faltantes se consideraron como ausentes al azar (NMAR en inglés), se rellenaron con un valor negativo (-1,000 para esta cohorte). Luego, se calculó una distancia de similitud del paciente usando la distancia euclidiana entre estos valores de tiempo hasta el evento para cada par de pacientes. Se construyó un agrupamiento jerárquico aglomerativo usando un enlace completo, la distancia entre los dos puntos más alejados en dos grupos. La selección del número apropiado de grupos se realizó a través de un análisis de estabilidad.

3. Resultados

Nuestros esfuerzos de minería de datos revelaron la información epidemiológica perteneciente a este hospital del IMSS, como se muestra en la Tabla 2. En esta cohorte, todos los pacientes fueron mujeres. La prevalencia más alta de cáncer estuvo dentro del grupo etario de 50 a 59 años. Hubo 207 pacientes (51%) diagnosticados con estadios tempranos de cáncer (etapas I y II). Sólo a 30 pacientes (7%) se les estimó el subtipo de cáncer de mama con la prueba de IHQ. De todos los pacientes, 18 pacientes (4%) fueron menores a 40 años. En este hospital 23 pacientes (6%) tuvieron una puntuación BIRADS de 2 o menor, lo que significa que no deberían haber sido enviados a la instalación de atención secundaria. En estos casos, los pacientes habían tenido investigaciones de biopsias externas en hospitales que no pertenecían al IMSS (generalmente privados) y decidieron continuar su atención para el tratamiento de seguimiento en los servicios de oncología del IMSS.

Tabla 2. Características clínicas de la cohorte y el estado de receptores para los pacientes con IHQ

**Características**	**Total (%)**	**Con pruebas de IHQ (%)**
**General**	**N = 402**	**N = 30**
Sexo		
Femenino	402 (100%)	30 (100%)
Masculino	0	0
Grupo etario		
<40	18 (4%)	3 (10%)
40-49	62 (15%)	16 (53%)
50-59	174 (43%)	7 (23%)
60-69	127 (32%)	4 (13%)
70+	21 (5%)	0
BIRADS		
0,1,2	23 (6%)	18 (60%)
3	203 (50%)	7 (23.3%)
4,5,6	162 (40%)	4 (13.3%)
desconocido	14 (3%)	1 (3.3%)
Estadío del tumor		
I	85 (21%)	2 (7%)
II	122 (30%)	12 (40%)
III	84 (21%)	12 (40%)
IV	19 (5%)	3 (10%)
desconocido	92 (23%)	1 (3%)
Pacientes sin prueba de IHQ	372 (93%)	0
Pacientes con prueba de IHQ	30 (7%)	30 (100%)
RE		
positivo	n.a.	17 (57%)
negativo		11 (37%)
desconocido		2 (7%)
RP		
positivo	n.a.	16 (53%)
negativo		12 (40%)
desconocido		2 (7%)
HER2		
positivo	n.a.	5 (17%)
negativo		23 (77%)
desconocido		2 (7%)
Otras pruebas de IHQ		
Ki67, P53	n.a.	1 (3%)
desconocido		29 (97%)
Subtipo acorde al Consenso Mexicano [4]	n.a.	

Luminal A	14 (47%)
Luminal B	3 (10%)
Tipo basal / Triple negativo	9 (30%)
HER2	2 (7%)
No hubo suficiente información	2 (7%)

Monitoreo Digital

Las trayectorias de los pacientes se segmentaron finamente como se muestra en la Figura 1, lo que permite el análisis digital (y por lo tanto la optimización). Cada paciente se representa en un panel (rectángulo) con barras de colores, indicando los eventos que experimentó un paciente en el hospital del IMSS. Cada fila representa un año de tratamiento de seguimiento para ese paciente, que puede ser de una a cuatro filas (porque el lapso máximo de tiempo de seguimiento fue de tres años y cinco meses). La longitud de cada barra de color representa el tiempo entre la ocurrencia de un evento y el evento que lo precedió. Aunque los eventos no ocurren continuamente, sino que ocurren en puntos individuales (por ejemplo, una visita al paciente, obteniendo los resultados de una radiografía), la representación visual que se muestra en la Figura 1 proporciona una idea de cuánto tiempo se requiere para cada acontecimiento, suponiendo que no ocurrió otra cosa al mismo tiempo.

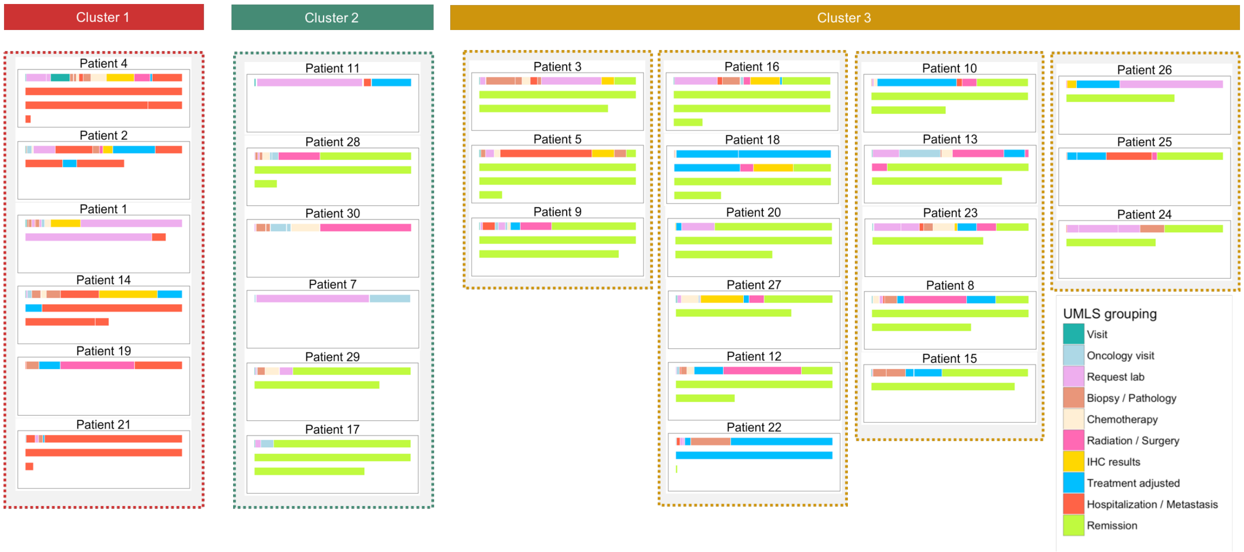

Figura 1. Gráficos de trayectoria médica de la cohorte con pruebas de IHQ. Cada paciente está representado por un rectángulo, con filas que representan cada año de seguimiento. Los eventos están codificados por colores de acuerdo con el tipo de evento, y la longitud de la barra representa la duración entre la ocurrencia de un evento y el evento que lo precedió.

Tiempo de respuesta

La minería de datos en esta cohorte identificó el tiempo de respuesta para las pruebas de IHQ, como se muestra en la Figura 2. Solo 20 pacientes tuvieron información en sus expedientes clínicos relacionada con el momento de las pruebas de IHQ. Aunque este servicio se menciona en los expedientes clínicos del IMSS, se subcontrata a un laboratorio privado. Hubo una gran variación en torno a dos variables clave: a) el tiempo de respuesta para obtener

resultados, con un tiempo promedio de 56 días (95% I.C. 36 - 77 días); y b) el tiempo transcurrido entre la solicitud de pruebas de IHQ en relación con el primer día de tratamiento de seguimiento en el hospital, con un tiempo promedio de 117 días (95% I.C. 80 - 154 días). En general, para los 30 pacientes con pruebas de IHQ, a 17 pacientes (56.7%) se les ajustaron sus tratamientos y 13 pacientes (43.3%) se les confirmaron sus tratamientos.

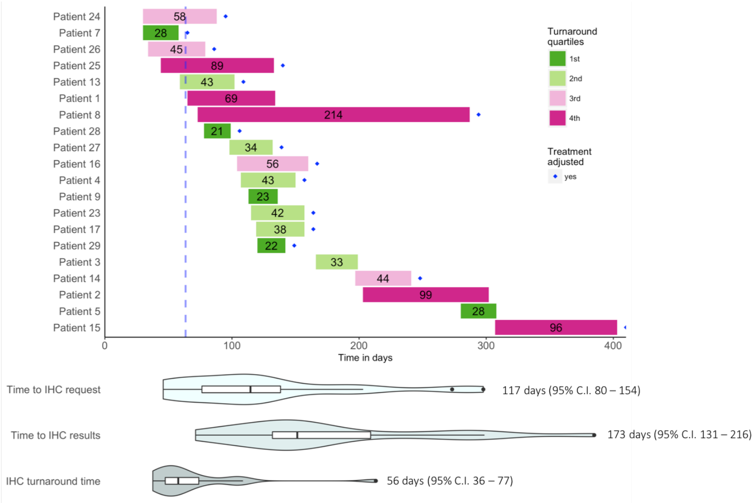

Figura 2. Tiempos de respuesta para las pruebas de IHQ. El tamaño de la barra indica el tiempo en días desde la solicitud de resultados para la prueba IHQ hasta la obtención de los mismos. Para cada paciente, el tiempo inicial es el primer día de tratamiento de seguimiento en el hospital. El color de la barra indica el cuartil dentro del cual los datos se encuentran en la distribución. La marca de diamante indica que el tratamiento se ajustó, de acuerdo con los protocolos, después de obtener los resultados de IHQ. La línea discontinua azul indica el primer cuartil por el tiempo que tomó antes de que se enviara una solicitud de IHC. Los diagramas de violín con bigotes (equivalentes a un diagrama de caja con una estimación de densidad de probabilidad) proporcionan una visión general del tiempo de respuesta necesario para las pruebas de IHQ en tres categorías: a) desde el primer día en el hospital para solicitar; b) desde el primer día en el hospital hasta obtener los resultados de IHQ; y c) de la solicitud de IHQ a la obtención de resultados.

4. Discusión

En México, el cáncer de mama continúa siendo un problema de salud pública, a pesar de que cada vez se realizan más investigaciones sobre sus cambios y características genómicas. Aunque nuestro estudio investigó una pequeña cohorte de pacientes, la prevalencia y las características epidemiológicas de nuestra cohorte son similares a las que se habían estimado previamente para el país (en cuanto a la edad y el estadio) [11]. Nuestro estudio investiga el uso de herramientas digitales de minería de datos para evaluar el porcentaje de pacientes que tienen pruebas de IHQ y la oportunidad en el uso de dichas. Esta herramienta puede usarse para proveer información a los administradores de hospitales y funcionarios de salud pública.

Sobre el valor terapéutico de las pruebas de IHQ. A pesar del pequeño número de pacientes con IHQ, los resultados sugieren la utilidad de estas pruebas en la toma de decisiones terapéuticas y en la determinación del pronóstico del paciente. En el hospital incluido en este estudio, las decisiones terapéuticas se toman en función de los resultados clínicos, quirúrgicos e histológicos, como la apariencia física, el tamaño del tumor, la invasión linfática, la necrosis tumoral, el tipo histológico, el grado de diferenciación y la cantidad de ganglios linfáticos afectados. Este estudio respalda la importancia terapéutica de la inmunofenotipificación (el uso de pruebas de IHQ para la subtipificación de pacientes con cáncer), como se ha demostrado ampliamente en otros estudios centrados en poblaciones en entornos de altos ingresos. Estos resultados deben considerarse y abordarse cuidadosamente en los planes de tratamiento creados por el equipo de atención multidisciplinaria. Es posible ajustar el tratamiento para mejorar el pronóstico del pacientes con la incorporación de información sobre el estado del receptor, el régimen de quimioterapia tradicional (FEC) recomendado por el protocolo IMSS, compuesto por 5-fluorouracilo, epirrubicina y ciclofosfamida.

La importancia terapéutica de la inmunofenotipificación (el uso de pruebas de IHQ para la clasificación de pacientes con cáncer) debe ser cuidadosamente considerada y evaluada en los planes de tratamiento que realizan los oncólogos. El esquema de quimioterapia tradicional (FEC) recomendado por el protocolo IMSS, compuesto por 5-fluorouracilo, epirrubicina y ciclofosfamida, podría ajustarse según las pruebas de biomarcadores de IHQ.

Los pacientes con estrógeno positivo (RE +) y / o progesterona positiva (RP +) podrían beneficiarse de la terapia antihormonal o endocrina para evitar recurrencia, ya sea inhibidores de la aromatasa para bloquear la producción de estrógeno (por ejemplo, anastrozol, letrozol o exemestano) o moduladores selectivos de los receptores de estrógeno (MSRE), que interfieren con la capacidad del estrógeno para estimular el crecimiento de las células mamarias (por ejemplo, tamoxifeno o toremifeno).

Adicionalmente, los pacientes con HER2 positivo (HER2 +), en los que los tumores tienden a crecer y diseminarse más agresivamente, podrían beneficiarse de una terapia dirigida que podría bloquear HER2 (por ejemplo, trastuzumab o pertuzumab). El uso de la hibridación *in situ* (ISH, en inglés), en lugar de la IHQ, se puede utilizar para determinar el estado de HER2 con una concordancia general con la IHQ, y puede ser más beneficioso utilizar ambos [19]. Además, el estado de HER2 se ha incorporado con éxito en la práctica médica para guiar las decisiones de tratamiento para los pacientes con cáncer de mama. De hecho, la Sociedad Estadounidense de Oncología Clínica / Colegio de Patólogos Estadounidenses (ASCO / CAP, en inglés) actualizaron sus directrices de 2013 para designar a más pacientes como elegibles para la terapia con trastuzumab, de acuerdo con las pruebas de ISH e IHQ [20]. También las pautas estratificadas por recursos de BHGI recomiendan el uso de trastuzumab en el nivel mejorado debido al alto costo del medicamento, así como también por su disponibilidad. El cuadro básico de medicamentos del IMSS [21] reporta al trastuzumab como un fármaco disponible para cualquier paciente con HER + positivo en el país.

Sobre la oportunidad en el tiempo de las pruebas. Las pruebas oportunas de IHQ, entendidas como la disponibilidad de los resultados de IHQ antes del comienzo de cualquier tratamiento, son de importancia crítica. Para el hospital del IMSS en este estudio, los resultados de IHQ generalmente no fueron oportunos, pero cuando estuvieron disponibles desencadenaron una respuesta del equipo de oncología clínica para ajustar los tratamientos de los pacientes. La amplia dispersión de tiempo para solicitar y el tiempo de respuesta demuestra la falta de estandarización de este proceso. Muchos factores podrían haber contribuido a la demora en las pruebas de IHQ, que incluyen: incumplimiento por parte del paciente de las citas; saturación de los servicios del hospital, lo que resulta en citas demoradas; problemas logísticos entre el IMSS y el servicio externo de laboratorio de IHQ; y problemas administrativos dentro del hospital que resultan en un seguimiento inadecuado de las pruebas y los resultados.

Sobre la necesidad de mejorar las guías de práctica clínica. Es importante establecer un cronograma apropiado para llevar a cabo pruebas de IHQ de forma oportuna. Por ejemplo, en los Estados Unidos, la guía del Colegio Americano de Patólogos (CAP, en inglés) sobre el tiempo de respuesta para las pruebas de biopsias es de alrededor de dos días en el 90% de los casos [22]. De hecho, la guía conjunta sobre las pruebas HER2 de la Sociedad Estadounidense de Oncología Clínica (ASCO) y CAP recomienda informar a los pacientes sobre el tiempo de respuesta esperado [23]. En Australia, un estudio reveló que el tiempo promedio de respuesta fue entre 4-5 días [24]. En Arabia Saudita, un estudio mostró que el 24% de los casos quedan fuera del tiempo de respuesta recomendado por CAP [25]. La Sociedad Europea de Oncología Médica (ESMO, en inglés) publicó una encuesta en 24 países europeos donde el tiempo de respuesta era de 10 días o menos para el 89% de los laboratorios [26]. En el Reino Unido, el Colegio Real de Patólogos recomienda el uso de indicadores de rendimiento clave para tener un diagnóstico histopatológico dentro de los siete días de la biopsia en el 90% de los casos [27].

Dada su disponibilidad de recursos, las guías de práctica clínica en los países desarrollados no tienen una recomendación sobre el tiempo máximo de respuesta que podría ser efectivo en la trayectoria de tratamiento de un paciente. Más importante aún, no abordan el tiempo transcurrido entre el diagnóstico inicial, las pruebas moleculares y la selección del tratamiento. En México, las guías de práctica clínica para el cáncer de mama recomiendan realizar pruebas de RE, RP y HER2 como parte del estudio de histopatología, pero no proporcionan una guía con respecto al tiempo de respuesta. El IMSS mantiene un manual de procedimientos médicos, que incluye diez indicadores clave para la detección, el diagnóstico y el tratamiento del cáncer de mama [28]. Este manual mide el tiempo máximo para diagnóstico en un período de 30 días, incluidas las imágenes de mamografía y los resultados del informe de histopatología. Además, la institución también mide el tiempo de inicio de tratamiento medido desde la fecha del diagnóstico, lo que debe lograrse dentro de un período de 21 días. Al reconocer las limitaciones de recursos del IMSS, la implementación de una política de indicadores clave relacionada con las pruebas de IHQ podría reducir significativamente los tiempos de entrega.

Sobre la necesidad de expedientes clínicos electrónicos (ECE) precisos. La información faltante fue común en todos los expedientes en papel y electrónicos de nuestro estudio. Los médicos y el personal administrativo del hospital del

IMSS todavía se están acostumbrando a la novedosa implementación del ECE, que tuvo un impacto en nuestra capacidad para caracterizar mejor esta cohorte. En ausencia de un registro de cáncer, nuestra mejor estimación de la enfermedad es el censo institucional. A medida que los hospitales en México y en otros lugares continúan siendo cada vez más electrónicos, existe la necesidad de desarrollar mejores herramientas de software para analizar la información obtenida, incluyendo el procesamiento de lenguaje natural médico y aplicaciones de aprendizaje automático.

Sobre la necesidad de monitorear el porcentaje de pacientes que se someten a la prueba. El monitor que se muestra en la Figura 1 proporciona rápidamente una descripción general del historial del paciente de la atención hospitalaria; mientras que la Figura 2 muestra información detallada del tiempo de retorno de los resultados. Con más trabajo en la interfaz de usuario y adecuada validación, imaginamos una herramienta que eventualmente podría representar una valiosa ayuda visual para los administradores de hospitales, proporcionando una visión general rápida de los pacientes atendidos en su hospital, que podría usarse como una herramienta de toma de decisiones.

Requiere atención especial el bajo porcentaje (7.4%) de pacientes que se observan que fueron evaluados con pruebas de IHQ. Una preocupación común de los estudios de revisión retrospectiva de registros médicos es el sub-reporte. La cantidad de información registrada en el hospital incluido en nuestro estudio puede mejorarse mediante la capacitación del personal clínico y administrativo sobre la importancia de solicitar y reportar las pruebas de IHQ. En ausencia de un registro de cáncer, nuestra mejor estimación de la enfermedad es el censo institucional, que no registra los resultados de las pruebas de IHQ. A medida que los hospitales en México y en otros lugares continúan siendo cada vez más electrónicos, es necesario desarrollar mejores herramientas de software para facilitar el registro y análisis de información clínica, incluido el procesamiento del lenguaje natural médico y aplicaciones de aprendizaje automático.

5. Conclusiones

Los eventos adversos en la trayectoria de un paciente oncológico, que pueden incluir hospitalización relacionada con el cáncer, recurrencia del tumor o metástasis, son extremadamente costosos para el sistema de salud. El uso de la prueba de IHQ demostró en nuestro estudio ser de ayuda con la selección del tratamiento preciso para los pacientes (ya sea ajustando el tratamiento o confirmándolo). El momento en que se realiza la prueba de IHQ es de importancia crítica si queremos influir en un mejor pronóstico.

Hemos revelado en esta cohorte que el uso de pruebas oportunas de IHQ se asocia con efectos terapéuticos beneficiosos en pacientes con cáncer de mama. Los objetivos de cualquier sistema de salud deben ser la identificación de eventos tempranos que puedan tener un impacto en los eventos futuros en la trayectoria del tratamiento de un paciente oncológico. En entornos con recursos limitados, es importante no solo considerar alternativas a diagnósticos más costosos (por ejemplo, pruebas genómicas), sino también incorporar los aspectos normativos y logísticos de la implementación de estas pruebas.

El IMSS debe enfrentar el importante desafío de continuar mejorando sus tiempos de respuesta, lo que debería tener un impacto positivo en el pronóstico de sus pacientes. A medida que el sistema de salud mexicano continúa la transición de la atención reactiva a la preventiva, la necesidad de más pruebas de IHQ en cáncer de mama y otras enfermedades permitirá un mayor desarrollo del monitoreo digital del paciente.

Declaraciones

Aprobación Ética

Esta investigación fue revisada y aprobada por el Comité Nacional de Investigación Científica y el Comité Nacional de Ética, ambos del Instituto Mexicano del Seguro Social (IMSS), con el número de protocolo R-2017-785-010, y también por el Comité Local de Investigación y Ética del IMSS bajo el número de protocolo específico R-2017-1602-14 HGR1 Charo, Michoacán. La Junta de Revisión Institucional de Stanford proporcionó una determinación de que la investigación no es considerada sobre sujetos humanos, bajo el protocolo electrónico: 40704. Los investigadores solo recibieron acceso a datos no identificables bajo las consideraciones de los estatutos mexicanos aplicables sobre investigación de salud, privacidad del paciente y expedientes clínicos electrónicos. No se requirió consentimiento informado.

Disponibilidad de datos

Los datos pertenecen al Instituto Mexicano del Seguro Social (IMSS), a través de su Delegación estatal de Michoacán. La Coordinación de Investigación en Salud del IMSS puede otorgar acceso a esta información caso por caso a los investigadores que obtengan las aprobaciones necesarias del Comité Nacional de Investigación Científica y del Comité Nacional de Bioética del IMSS.

Conflicto de intereses

RAR declara ser el delegado estatal del IMSS en Michoacán, supervisando el hospital mencionado en este estudio. Los autores restantes no declaran tener conflictos de intereses.

Fondos de investigación

La investigación reportada en esta publicación fue parcialmente respaldada por fondos institucionales de cada institución participante, ya sea el Instituto Mexicano del Seguro Social (IMSS) o la Universidad Stanford. Además, ALP y CDB fueron parcialmente respaldados por los siguientes fondos: U01 HG007436-04 de la base de datos de variaciones genómicas clínicamente relevantes del Instituto Nacional de Investigación del Genoma Humano (NHGRI); y U01 FD004979 del Centro de Excelencia en Ciencia Regulatoria e Innovación (CERSI) de la Administración de Alimentos y Medicamentos (FDA) de la Universidad de California en San Francisco y la Universidad Stanford. Los fondos de investigación no tuvieron ningún papel en el diseño del estudio, la recopilación y el análisis de datos, la decisión de publicar o la preparación del manuscrito.

Contribuciones de los autores

ALP diseñó el estudio. MFRM revisó notas médicas. MFRM y ALP realizaron el análisis de datos. ASB, SNS, RAR, CAA, SMFV y CDB proporcionaron interpretación de los resultados. ALP redactó el manuscrito y todos los autores contribuyeron críticamente, leyeron, revisaron y aprobaron la versión final.

Agradecimientos

Los autores desean agradecer a la Dra. Katie Kanagawa, por su valioso apoyo en la edición de este manuscrito en inglés.

Referencias

1 Lips EH, Mulder L, de Ronde JJ, *et al.* Breast cancer subtyping by immunohistochemistry and histological grade outperforms breast cancer intrinsic subtypes in predicting neoadjuvant chemotherapy response. *Breast Cancer Res Treat* 2013; **140**: 63–71.

2 Won JR, Gao D, Chow C, *et al.* A survey of immunohistochemical biomarkers for basal-like breast cancer against a gene expression profile gold standard. *Mod Pathol* 2013; **26**: 1438–50.

3 Abu-Khalf M, Pusztai L. Influence of genomics on adjuvant treatments for pre-invasive and invasive breast cancer. *Breast* 2013; **22 Suppl 2**: S83–7.

4 Cardenas Sanchez J, Bargallo Rocha JE, Erazo Valle A, Poitevin Chacon A, Valero Castillo V, Perez-Sanchez V. [Consenso Mexicano sobre diagnóstico y tratamiento del cáncer mamario], 6 edn. 2015.

5 Secretaria de Salud de Mexico. [Diagnóstico y Tratamiento de la Patología Mamaria Benigna en Primer y Segundo Nivel de Atención]. 2009; : 1–66.

6 Secretaria de Salud de Mexico. [Prevención, Tamizaje y Referencia Oportuna de Casos Sospechosos de Cáncer de Mama en el Primer Nivel de Anteción]. 2011.

7 Secretaria de Salud de Mexico. [Diagnóstico y Tratamiento del Cáncer de Mama en Segundo y Tercer Nivel de Atención]. 2012; : 1–102.

8 Lara-Medina F, Perez-Sanchez V, Saavedra-Perez D, *et al.* Triple-negative breast cancer in Hispanic patients: high prevalence, poor prognosis, and association with menopausal status, body mass index, and parity. *Cancer* 2011; **117**: 3658–69.

9 Robles-Castillo J, Ruvalcaba-Limón E. [Cáncer de mama en mujeres mexicanas menores de 40 años]. *Ginecologia y Obstetricia de Mexico* 2011; **79**: 482–8.

10 Villarreal-Garza C, Alvarez-Gomez RM, Perez-Plasencia C, *et al.* Significant clinical impact of recurrent BRCA1 and BRCA2 mutations in Mexico. *Cancer* 2015; **121**: 372–8.

11 Mohar-Betancourt A, Reynoso-Noveron N, Armas-Texta D, Gutierrez-Delgado C, Torres-Dominguez JA. Cancer Trends in Mexico: Essential Data for the Creation and Follow-Up of Public Policies. *Journal of Global Oncology* 2017; : JGO.2016.007476–9.

12 Shaaban AM, Purdie CA, Bartlett JMS, *et al.* HER2 testing for breast carcinoma: recommendations for rapid diagnostic pathways in clinical practice. *J Clin Pathol* 2014; **67**: 161–7.

13 Anderson BO, Yip C-H, Smith RA, *et al.* Guideline implementation for breast healthcare in low-income and middle-income countries: overview of the Breast Health Global Initiative Global Summit 2007. Wiley Subscription Services, Inc., A Wiley Company, 2008: 2221–43.

14 Carlson RW, Scavone JL, Koh W-J, *et al.* NCCN Framework for Resource Stratification: A Framework for Providing and Improving Global Quality Oncology Care. *J Natl Compr Canc Netw* 2016; **14**: 961–9.

15 Chuang LT, Temin S, Berek JS. Management and Care of Women With Invasive Cervical Cancer: American Society of Clinical Oncology Resource-Stratified Clinical Practice Guideline Summary. *J Oncol Pract* 2016; **12**: 693–6.

16 World Health Organization. Executive Summary. National Cancer Control Programmes. Policies and Managerial Guidelines. 2012.

17 Instituto Nacional de Estadística y Geografía. [Encuesta Intercensal 2015]. 2015.

18 Instituto Mexicano para la Competitividad. [Indice de Competitividad Estatal 2016]. 2016; : 1–202.

19 Solomon JP, Dell'Aquila M, Fadare O, Hasteh F. Her2/neu Status Determination in Breast Cancer: A Single Institutional Experience Using a Dual-Testing Approach With Immunohistochemistry and Fluorescence In Situ Hybridization. *Am J Clin Pathol* 2017; **147**: 432–7.

20 Lim TH, Lim AST, Thike AA, Tien SL, Tan PH. Implications of the Updated 2013 American Society of Clinical Oncology/College of American Pathologists Guideline Recommendations on Human Epidermal Growth Factor Receptor 2 Gene Testing Using Immunohistochemistry and Fluorescence In Situ Hybridization for Breast Cancer. *Arch Pathol Lab Med* 2016; **140**: 140–7.

21 Instituto Mexicano del Seguro Social. [Cuadro Basico de Medicamentos. Oncologia]. 2017.

22 Novis DA, Zarbo RJ, Saladino AJ. Interinstitutional comparison of surgical biopsy diagnosis turnaround time: a College of American Pathologists Q-Probes study of 5384 surgical biopsies in 157 small hospitals. *Arch Pathol Lab Med* 1998; **122**: 951–6.

23 Wolff AC, Hammond MEH, Hicks DG, *et al.* Recommendations for Human Epidermal Growth Factor Receptor 2 Testing in Breast Cancer: American Society of Clinical Oncology/College of American Pathologists Clinical Practice Guideline Update. *J Clin Oncol* 2013; **31**: 3997–4013.

24 Morey AL, Brown B, Farshid G, *et al.* Determining HER2 (ERBB2) amplification status in women with breast cancer: final results from the Australian in situ hybridisation program. *Pathology* 2016; **48**: 535–42.

25 Alshieban S, Al-Surimi K. Reducing turnaround time of surgical pathology reports in pathology and laboratory medicine departments. *BMJ Qual Improv Rep* 2015; **4**: u209223.w3773.

26 Boleij A, Tembuyser L, Taylor A, *et al.* PD-015A survey on current RAS-mutation testing practices in Europe. *Ann Oncol* 2015; **26**: iv105–5.

27 Pathologists TRCO. Key Performance Indicators in Pathology. 2013; : 1–23.

28 Instituto Mexicano del Seguro Social. [Manual Metodológico de Indicadores Médicos 2016]. 2016; : 1–452.
